# Analysis of Geometrical Accuracy and Surface Quality of Threaded and Spline Connections Manufactured Using MEX, MJ and VAT Additive Technologies

**DOI:** 10.3390/ma17215143

**Published:** 2024-10-22

**Authors:** Marcin Sarzyński, Kamila Chudzik, Paweł Panek, Bartłomiej Sarzyński, Małgorzata Zaborniak

**Affiliations:** 1 Faculty of Mechatronics and Aerospace, Military University of Technology, 2 Gen. S. Kaliskiego Street, 00-908 Warsaw, Poland; 2 Faculty of Mechanical Engineering and Aeronautics, Rzeszow University of Technology, Powstancow Warszawy 8 Str, 35-959 Rzeszow, Poland; kamliachudzik4@gmail.com (K.C.); panekp94@gmail.com (P.P.); mzab@prz.edu.pl (M.Z.); 3 Institute of Robots & Machine Design, Faculty of Mechanical Engineering, Military University of Technology, Gen. S. Kaliskiego St., 00-908 Warsaw, Poland; bartlomiej.sarzynski@wat.edu.pl

**Keywords:** mechanical engineering, additive manufacturing, photopolymer, material extrusion, mechanical joints, bolt joints, spline joints, PolyJet, SLA, FDM

## Abstract

This paper presents the process of manufacturing mechanical joint components using additive manufacturing (AM) techniques such as Material Extrusion (Fused Deposition Modelling (FDM)), Material Jetting (PolyJet), and Vat Photopolymerization (VAT)/Stereolithography (SLA). Using the PolyJet technique and a photopolymer resin, spline and threaded joint components were produced. For comparative analysis, the threaded joint was also fabricated using FDM and SLA techniques. PLA material was used for the FDM technique, while photopolymer resin was utilized for the SLA process. The components produced underwent a surface analysis to evaluate the accuracy of the dimensions in relation to the nominal dimensions. For the spline connection components, the dimensional deviations recorded by a 3D scanner ranged from −0.11 to +0.18 mm for the shaft and up to 0.24 mm for the sleeve. Measurements of screw and nut diameters showed the highest accuracy for screws produced using the PolyJet technique, while the nuts exhibited the best accuracy when fabricated with the SLA method. The profile of the screw threads using a contour gauge revealed the most accurate thread profile on the screw manufactured with the PolyJet technique.

## 1. Introduction

In the fast-paced technology sector of today, the quest for innovative manufacturing techniques is essential for improving efficiency and lowering expenses. [1]. These principles form the foundation of the term Industry 4.0, where the human factor is being replaced by autonomous systems and new production technologies [2]. A significant aspect of this domain includes spline and threaded connections, which find extensive application in mechanics, machinery construction, and the automotive sector [3,4]. Traditional methods of manufacturing these connections, despite their effectiveness, are often time-consuming and costly, which motivates the search for new, more efficient production technologies [5,6]. Additive manufacturing techniques, commonly referred to as 3D printing, present a potential solution in this regard [7]. Research on the use of additive manufacturing techniques in the production of various components, applicable in many fields, is one of the main topics discussed in the world of science and research [8]. This growing interest is evidenced by the steadily increasing number of publications focusing on components produced through additive methods [9].

Spline connections are classified as detachable form-fit joints and are most commonly used in mass production [10]. Their design facilitates the transmission of substantial torque forces, rendering them essential in various mechanical applications [11,12]. Traditional methods of producing splines are characterized by multiple production stages and high manufacturing costs. This process typically involves complex machining operations, such as milling, turning, and grinding, which require significant labor and time [13]. As a result, conventional methods of spline production are primarily used in mass production, where unit costs can be reduced through economies of scale [14]. In contrast, unit production, where these costs become excessively high, renders traditional methods economically unfeasible. Furthermore, many machines feature unique, specially designed spline connections that are often not readily available in the market. In cases of component damage, a swift restoration and reproduction of functionality are necessary. In such situations, additive manufacturing techniques, like rapid prototyping, can be advantageous [15].

The primary benefits of threaded connections include their established strength and the ease of disassembly without damaging the components involved. The production of screws and nuts through machining and subtractive processes is efficient for mass production; however, it involves a multi-stage process requiring multiple machines [16]. The main advantages of threaded connections are their well-documented strength and the ease of disassembly without damaging the connected components. The production of screws and nuts using machining and subtractive methods, while efficient for mass production, involves a multi-stage process that requires numerous machines [17]. The end of the 20th century introduced a new manufacturing technique—3D printing—which allows for the creation of physical models based on geometry designed in Computer-Aided Design (CAD) systems, bypassing many stages of traditional manufacturing processes [18]. In many instances, additive manufacturing methods can serve as a viable alternative for currently unavailable threaded connections. Recent years have seen a rapid development of additive manufacturing techniques such as 3D printing [19]. CAD techniques have become widely used in machine construction, particularly in the creation of prototypes and components with complex geometries [20,21,22,23,24]. 3D printing accelerates production processes by replacing conventional technologies that are both time-consuming and costly [25,26]. Additive manufacturing methods are applicable in both mass production and unit production, where they are used to produce specialized or customized parts [27]. One of the greatest advantages of 3D printing is the efficient use of building material and the relatively straightforward production process, which consists of three stages: preparing the 3D model, manufacturing, and simple post-processing, typically involving the removal of support material [28,29]. Additive manufacturing is particularly valuable in unit production of components that are impossible, significantly more expensive, or time-consuming to produce using conventional methods. 3D printing allows for the creation of complex geometries without the need for specialized tools, which significantly reduces production costs [30,31]. The prevailing trend in this domain includes Rapid Prototyping (RP), which is increasingly gaining traction in the market. This trend is largely driven by the adaptability of these technologies to create complex components in a relatively short timeframe and at low costs, partially replacing traditionally manufactured components.

This article aims to demonstrate the potential of 3D printing as a modern and efficient manufacturing tool that can significantly impact the development of prototype spline and threaded connection technologies, making them more accessible and cost-effective. Additionally, three additive manufacturing techniques using polymers were compared in terms of dimensional accuracy of the surfaces. The integration of additive technologies in the production of mechanical components not only reduces costs but also opens up new design possibilities, enabling the creation of more advanced and precise components.

## 2. Materials and Methods

The additive manufacturing process was carried out using three technologies:

- PolyJet;
- Stereolithography (SLA);
- Fused Deposition Modelling/Fused Filament Fabrication/Material Extrusion (FDM/FFF/MEX).

Spline connection components were produced exclusively using the PolyJet technique. For comparison of the three aforementioned methods, threaded connection components were produced using three different techniques. The PolyJet technique utilizes liquid resin and allows for the precise creation of high-accuracy objects. The printing was carried out using the Stratasys Objet 350 Connex3 (Stratasys, Eden Prairie, MN, USA). Stratasys RGD720 material was used for the research components, with Stratasys RGD706 as the support material. The same materials were used for the production of the threaded connection components. Model preparation for printing was carried out in the dedicated Objet Studio for Objet 350 Connex3 software by Stratasys, which automatically generated the necessary supports for correct 3D model fabrication. The “High Quality” mode was set to ensure the best print quality, and a matte finish was applied to the components. The construction time for four components was 4 h and 40 min, using 245 g of building material and 82 g of support material.

For the production of screws and nuts using the FDM technique, Prusa MK3 device from Prusa i3 (Prague, Czech Republic) was used. The FDM technique, also known as FFF or MEX according to ISO/ASTM 52900:2021, involves depositing successive layers of heated material through a nozzle to achieve the desired geometry [32]. Table 1 shows the parameters used in the fabrication process.

In this case, PLA (polylactic acid) material was used for the research components. The third technique used to produce threaded connections was SLA. SLA is an additive manufacturing process in which an object is created by selectively curing photopolymer resin, layer by layer, using an ultraviolet (UV) laser beam. The Peopoly Moai (U.S.) device and the Peopoly photopolymer resin were used for producing the research components.

**Figure 1:**
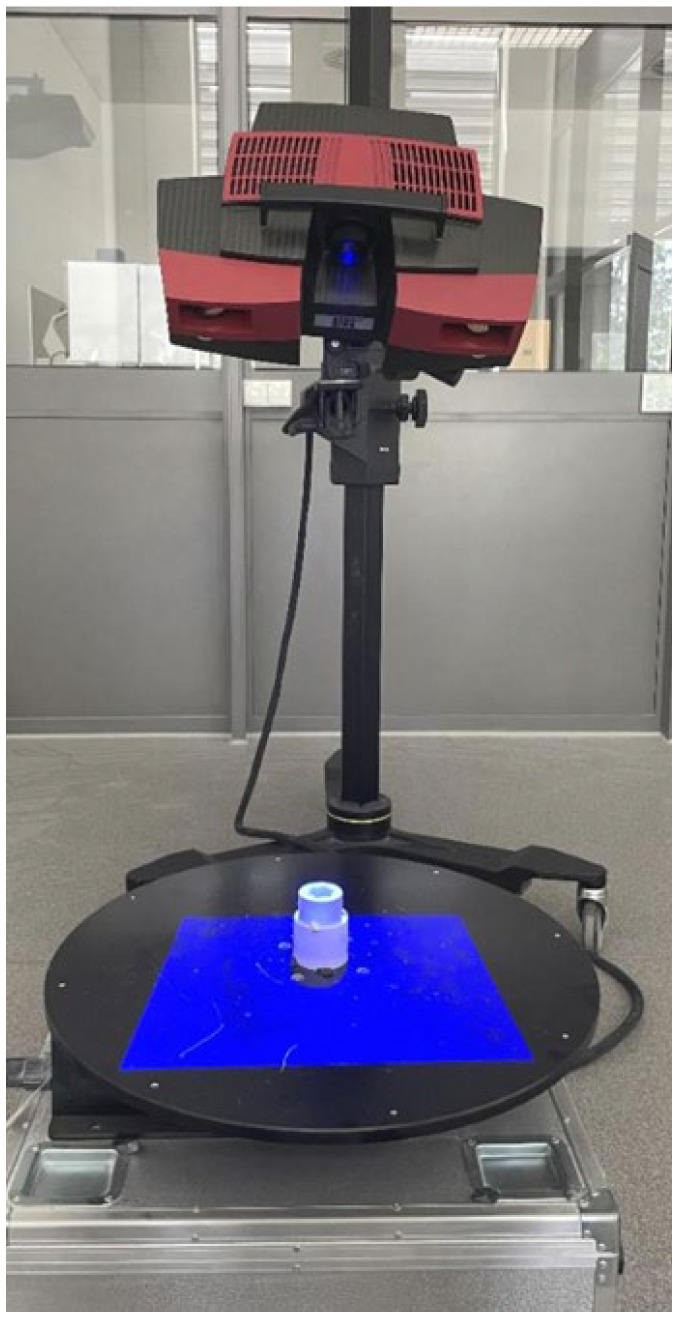
Measurement setup equipped with the ATOS Triple Scan II Blue Light 3D scanner.

**Figure 2:**
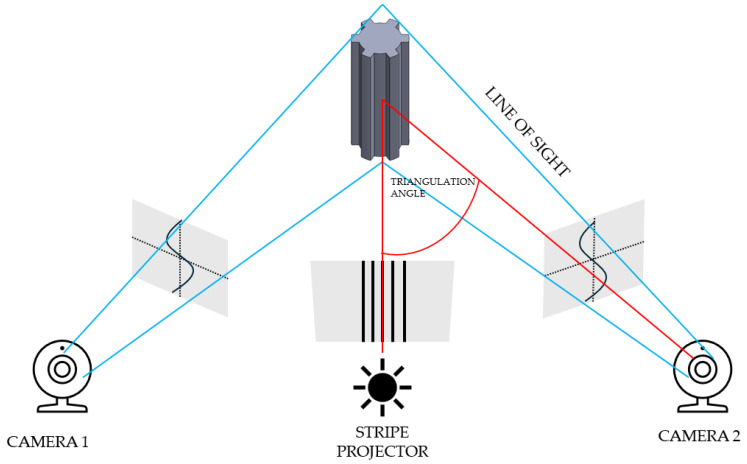
Principle of operation of a 3D scanner.

The first stage of geometric accuracy analysis focused on the spline connection components. Geometric accuracy measurements of the produced spline connection components were carried out using the ATOS Triple Scan II Blue Light optical 3D scanner (Zeiss, Oberkochen, Germany) presented in Figure 1. This device, which employs structured blue light technology, offers high-resolution, rapid measurements of three-dimensional coordinates. Accurate pixel-level coordinate capture is achieved through phase-shifting projection with two 5-megapixel cameras. This scanner supports production processes and is commonly used during design optimization. The ATOS optical system (Bezons, France) is also used for part inspection in industrial environments and in reverse engineering processes. The advantages of optical scanning systems by ZEISS INSPECT include automatic calibration checks during each measurement and quality control of stripe projections. A 3D scanner works by capturing the shape of an object using various techniques to generate a three-dimensional digital representation. The most common method is based on structured light or laser triangulation, where the scanner projects a light pattern onto the object’s surface. Sensors detect the deformation of the projected pattern or laser beam, and from this data, the scanner calculates the object’s geometry. Multiple scans from different angles are often taken to capture all aspects of the object. These scans are then merged into a single, complete 3D model using specialized software. The precision of the scanning depends on the type of technology used, with laser scanners typically offering higher accuracy than other methods. 3D scanning can also utilize time-of-flight (ToF) technology, where the scanner measures the time it takes for a light pulse to travel to the object and back. The resulting point cloud, a set of data points in 3D space, represents the object’s surface in great detail. This point cloud can be processed into a mesh or CAD model for further applications. 3D scanning is widely used in industries like manufacturing, design, and healthcare for prototyping, quality control, and reverse engineering. The schematic illustration showing the principle of operation of the 3D scanner is presented in Figure 2. The Triple Scan system allows for the digitization of dark or highly reflective surfaces and complex geometries. The standard measurement setup includes a measurement head, a data processing computer, and appropriate software. Table 2 presents the key parameters of the scanner used in this research.

**Figure 3:**
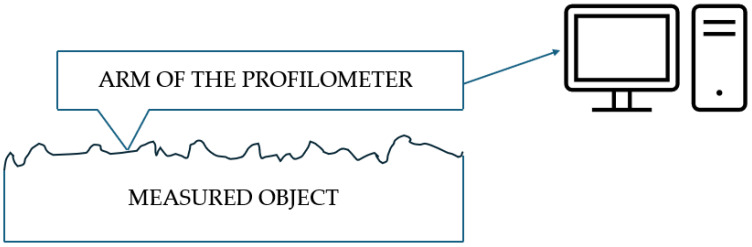
The diagram illustrating the operation of the contour graph.

To analyze the accuracy of the threaded connection components, the MarSurf XC 20 contour profiler by Mahr GmbH (Göttingen, Germany) was used. The diagram illustrating the operation of the contour graph is presented in Figure 3. A contour graph is an instrument used for precise surface profiling and measurement of three-dimensional objects. It operates by utilizing a stylus that follows the contour of the surface being analyzed. As the stylus traverses the surface, it converts the vertical movements into electrical signals, which are then recorded for further analysis. The data obtained allows for the generation of a detailed topographical map of the surface, showcasing features such as roughness and texture. This technology is widely employed in quality control, materials science, and engineering applications to assess and improve surface properties. The MarSurf XC 20 is a fundamental measurement station for assessing surface roughness and contours. It enables a precise evaluation of surface and contour parameters, which is crucial for analyzing the precision of threaded connection components. With its advanced software, the system allows for detailed measurements essential to assess the quality of printed components.

## 3. Results

### 3.1. Analysis of the Accuracy of Spline Connection

**Figure 4:**
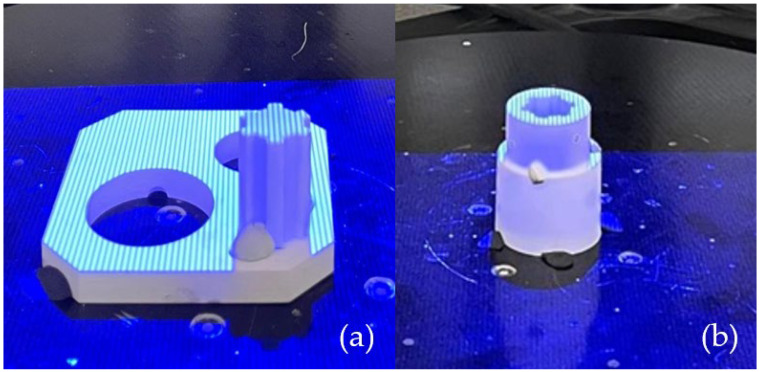
Discretization process: (**a**) shaft geometry; (**b**) sleeve geometry.

A set of spline connection components was produced using the PolyJet technique. The support material was removed using a pressure washer. The prints had a straw-colored appearance and featured a smooth surface. Before measurement, the surface of the object needed to be matting. To achieve this, a matting powder was applied to reduce surface reflectivity, with an antireflective layer thickness of 0.02 mm. The next preparatory step for accurate measurement involved applying measurement markers to the object. These circular markers, placed on the scanned object, serve as reference points on its surface. The prepared samples were subjected to the scanning process shown in Figure 4. The resulting models were saved in .stl format.

**Figure 5:**
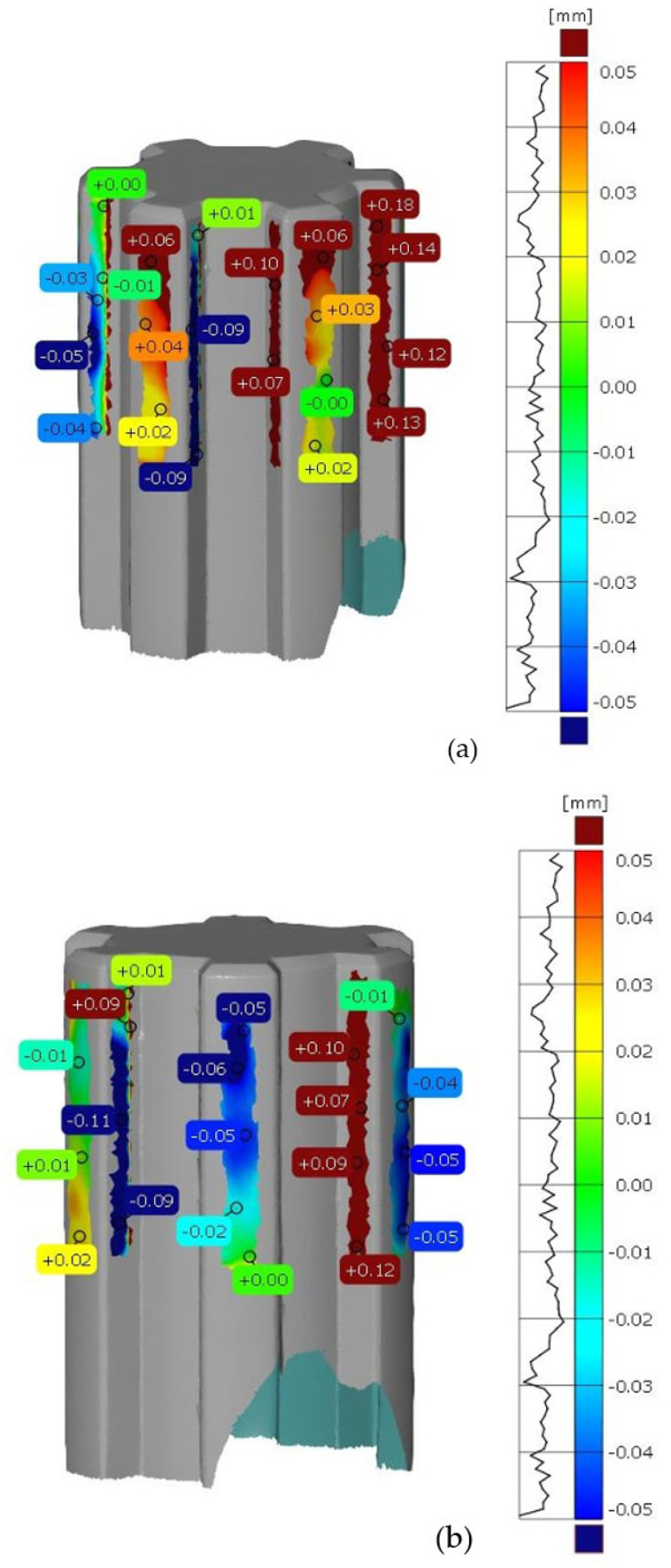
Deviation analysis between the reference model and the print. (**a**) front side of the shaft, (**b**) rear side of the shaft.

**Figure 6:**
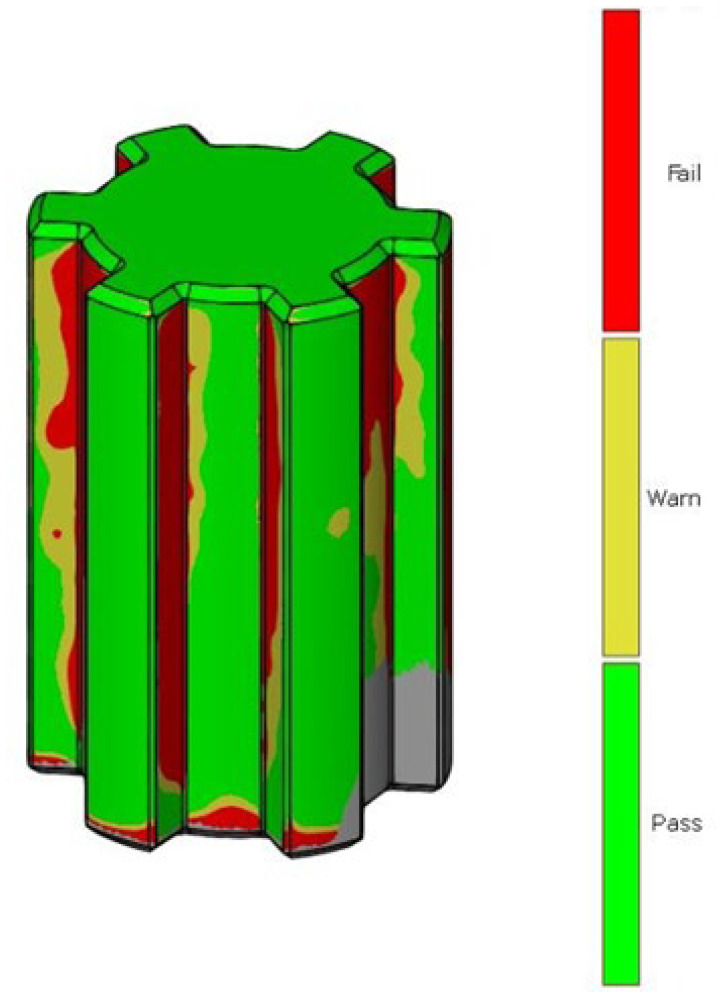
Dimensional tolerance analysis of shaft geometry.

After scanning the research components, a process was conducted to compare the geometric accuracy of the scans with reference models possessing nominal dimensions. The surfaces of the measured components were divided into the front and rear sides. Figure 5 presents the results of the deviation analysis between the reference model of the shaft and the print. The color scale on the right side of each image shows the deviation values in millimeters. Warm colors (red, yellow) indicate positive deviations, while cool colors (shades of blue) represent negative deviations. Green indicates areas with dimensions that match the reference model. Analysis of the measurement results shows that the deviation range for the printed component geometry is from −0.11 mm to +0.18 mm. Figure 6 shows the tolerance analysis for the entire component, with the tolerance limit set at 75% of the maximum deviation of 0.25 mm. Red indicates deviations greater than 0.625 mm, yellow represents the range between −0.625 mm and +0.625 mm, and green denotes no deviation. On the basis of the deviation distribution analysis, it can be observed that deviations are particularly present on the side surfaces of the spline connections.

**Figure 7:**
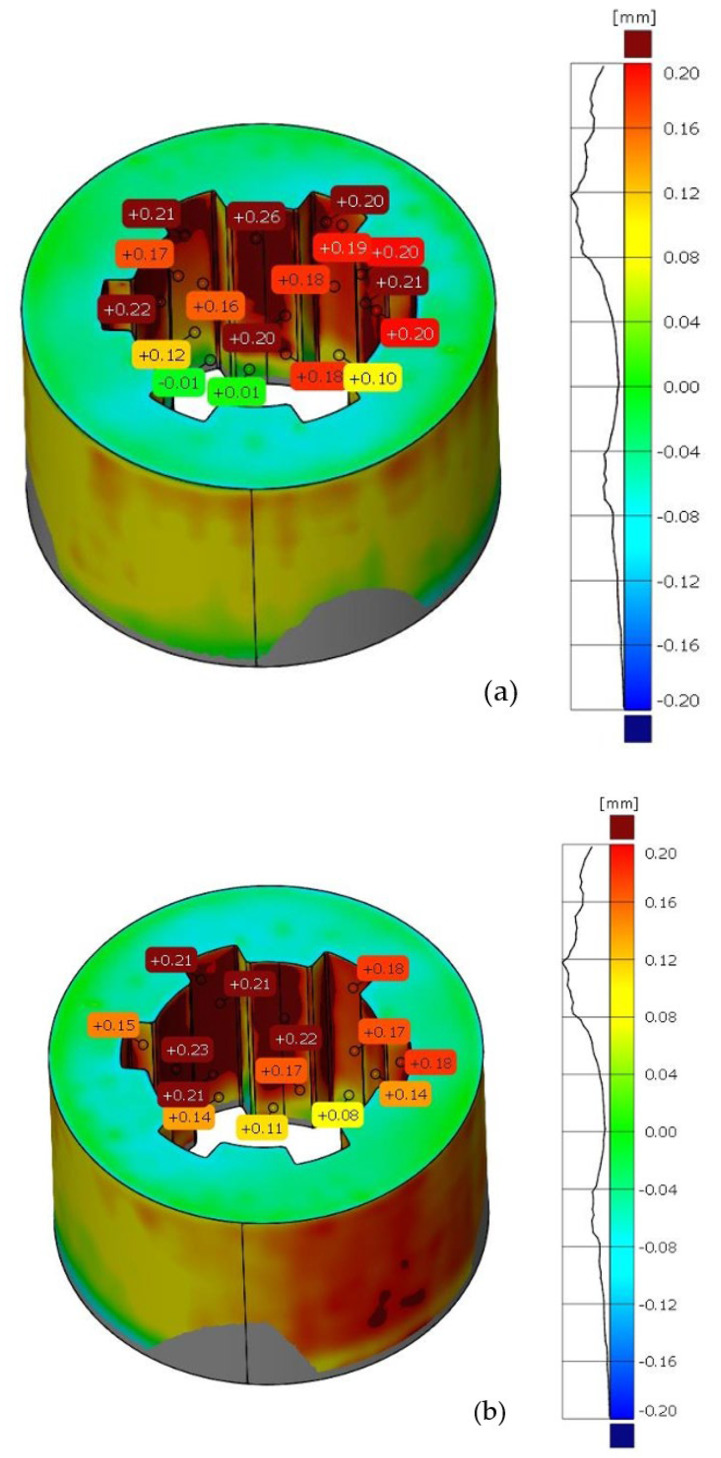
Deviation analysis between the reference model and the print. (**a**) Front side of the sleeve, (**b**) rear side of the sleeve.

**Figure 8:**
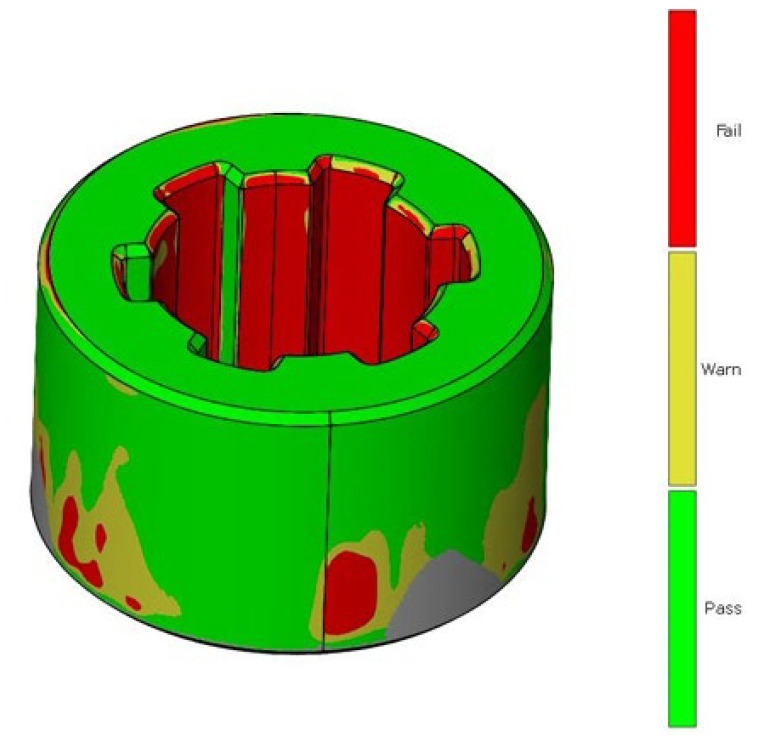
Dimensional tolerance analysis of sleeve geometry.

The same methodology was used for the analysis of deviations in the geometry of the sleeve. Figure 7 shows the deviation analysis between the reference model and the model obtained from the 3D scanning process of the additively manufactured sleeve. Based on the dimensional deviation analysis, it is observed that, unlike the shaft model, the deviations are exclusively positive. The smallest deviation is 0.02 mm, while the largest deviation is 0.24 mm. Deviations mainly occur on the side surface of the spline connections and at the geometric notch (the junction between the cylindrical geometry and the side surface of the spline). Figure 8 presents the tolerance analysis set at 0.75% of the maximum deviation of 0.25 mm. The image shows that the internal geometry of the spline exhibits a significant dimensional deviation.

### 3.2. Accuracy Analysis of Threaded Connections

**Figure 9:**
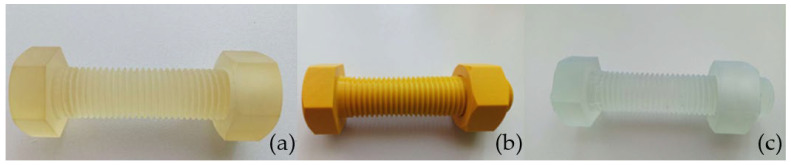
Screws and nuts are produced using the following techniques: (**a**) PolyJet, (**b**) FDM, and (**c**) SLA.

The accuracy analysis of the threaded connections was conducted based on models produced using PolyJet, FDM, and SLA techniques, as shown in Figure 9. For comparison and reference, measurements were also taken of a metal M30 × 120 grade 8.8 zinc-coated screw.

Measurements of the external thread diameter of screws and nuts were conducted. The obtained data are presented in Table 3. The reference screw has a dimension of 29.72 mm. Among all the printed screws, the one produced using the PolyJet technique was closest to this dimension at 29.73 mm. The screw produced using FDM was smaller than the reference screw by 0.20 mm, measuring 29.52 mm. The worst result was obtained by the screw produced using the SLA technique, which measured 28.70 mm, showing a difference of 1.02 mm from the reference screw.

Additionally, measurements of the nut diameters were performed. The reference nut achieved a diameter of 26.72 mm. The nut produced using the SLA technique was closest to this dimension, measuring 26.55 mm. The FDM technique was the second most accurate, with a dimension of 26.44 mm. The PolyJet technique demonstrated the worst dimensional accuracy, with the nut produced measuring 26.32 mm, resulting in a 0.40 mm difference from the reference nut.

After measuring the screws and nuts with a caliper, measurements were also carried out using a contourograph. Due to the nature and construction of the available device, measurements were performed only on screws. Internal thread measurements on nuts were not feasible due to their diameter. The measurements were carried out in two passes. The first pass was conducted with a small point collection step of 4 μm, meaning the program measured and recorded a point at this distance. The second pass used an increased step value of 10 μm. Each measurement resulted in a contour drawn in the program based on the measured points, reflecting the actual surface of the measured part.

**Figure 10:**
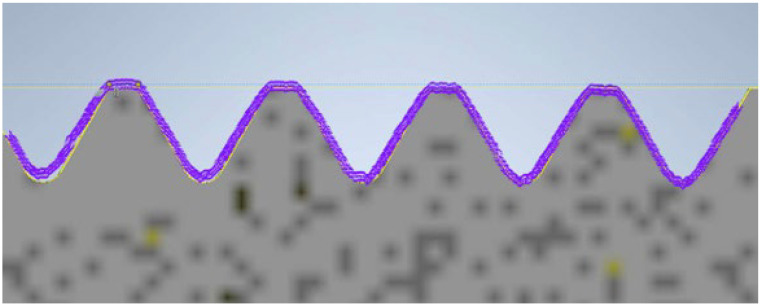
Thread profile of the screw produced using PolyJet technology overlaid on the profile of the reference screw (the gray profile represents the nominal profile, while the purple outline is the reading from the contour graph).

During the contour graph analysis, the first element examined was the screw manufactured using PolyJet technology. In Figure 10, the contour of the thread read by the contour graph is represented by the purple curve. At first glance, it does not deviate from the reference profile. The 3D model of the screw produced using PolyJet meets the requirements; the surface is smooth and accurate. Both the pitch and the thread angle are close to the values specified by the reference model.

**Figure 11:**
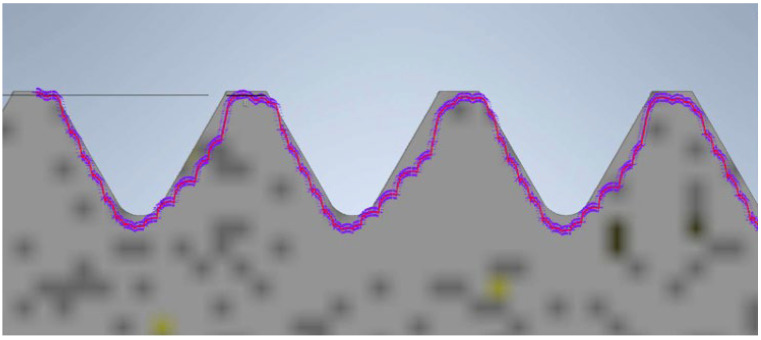
Thread profile of the screw produced using FDM technology overlaid on the profile of the reference screw (the gray profile represents the nominal profile, while the purple outline is the reading from the contour graph).

The next model of the screw and nut was produced using FDM technology. The view of the recorded profile overlaid on the reference profile is shown in Figure 11. In this case, the noticeable characteristic of material shrinkage of the printing process is evident. This results in a slight reduction in the diameter and thread surface. The thread surface is rough, and individual print layers are visible. Despite these inaccuracies, the nut produced using this technique fits onto the screw.

**Figure 12:**
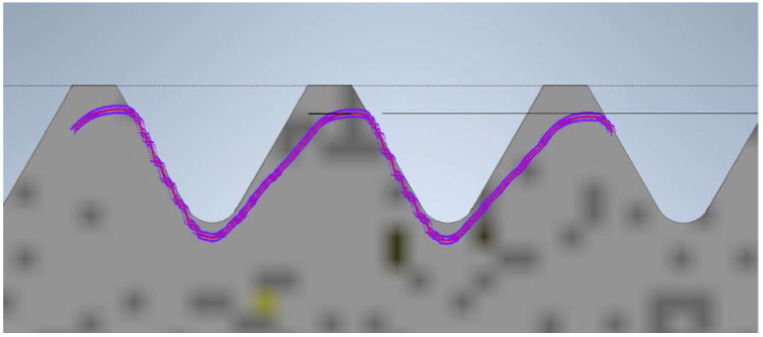
Screw thread profile produced using SLA technology overlaid on the profile of the reference screw (the gray profile represents the nominal profile, while the purple outline is the reading from the contour graph).

The final model is the screw and nut produced using SLA technology. The view of the contour graph results is shown in Figure 12, where the thread profile tilt aligned with the printing direction is visible. This is influenced by the cooling of the resin during the model creation. The profile overlaid on the theoretical outline is significantly reduced. This reduction is caused by substantial shrinkage of the resin. The profile exhibits a pronounced tilt in the direction of printing. The nut can be screwed onto the screw, but there is a noticeable play in one direction.

## 4. Discussion

In the first part of the work, scanning of spline connection elements was conducted, and an analysis of dimensional and geometric accuracy was performed. This study, carried out using ZEISS INSPECT Optical 3D software, revealed geometric deviations that occurred during the printing of the elements. When analyzing the results, it is important to consider that each intermediate process during manufacturing affects the accuracy of the measurement. The conversion of solid models to triangular meshes, the printing process itself, the method of support removal, and the calibration of the measurement setup all have a significant impact on measurement accuracy. The analyses show that most deviations are positive, especially around the notch outline. It is noticeable that in many cases, deviations reach values of around one-tenth of a millimeter. Excess material may result from an improperly executed rinsing process after the prototyping, leaving a thin layer of support material on the surface. It should also be noted that during optical scanning, an anti-reflective coating with a thickness of 0.02 mm was applied to the models [33]. During PolyJet prototyping, the most significant factor causing discrepancies is the material’s polymerization shrinkage. The research carried out in this work aimed to find a more cost-effective alternative to traditional spline manufacturing. Additively manufactured spline connection elements can be particularly useful in prototype construction due to their relatively low production cost compared to conventional methods.

Analyzing screw connections, the model most closely matching the expected result was produced using PolyJet technology. The elements had a smooth surface, and the measured profile almost matched the theoretical one. The thread profile had few discontinuities and its angle and pitch were close to the desired values, approximately 63° and 3.50–3.54 mm, respectively. The nut and bolt formed a fitting connection, with the nut screwing onto the standard bolt quite tightly. In the FDM method, layer transitions were unfortunately visible [34]. This roughness was noticeable in the report and in the profile [35]. Despite this, the nut was screwed onto the bolt. In SLA technology, the screw and nut fit together. The smooth surface of the print is a positive aspect. However, the report showed that the contour was clearly inclined in the direction of printing, with dimensional differences from the standard model reaching up to 1 mm [36].

From the above data, it can be concluded that Rapid Prototyping methods can be versatile for producing screw connections. However, it is essential to remember that creating a model according to standards will not yield the expected results. This is because standards describe the geometry of the tool used to cut threads. In additive methods, no tools are used to cut or form the outline. Therefore, some corrections are necessary. After refining parameters and dimensions, each of the mentioned technologies can be used to manufacture fastening elements. Proper model orientation during printing is crucial. For axisymmetric elements, the vertical printing orientation is best. All models for this work were printed in this orientation. All prints were successfully assembled, and most of them also fit with the standards. Among the models discussed in this work, PolyJet technology leads, similarly to the data in the articles [37,38,39]. The print demonstrated that RP technologies do not have significant issues recreating the screw thread.

In conclusion, this study mainly focused on a limited selection of materials for each additive manufacturing technique, highlighting the significant potential for further exploration of alternative materials. The dimensional accuracy of produced components, such as screws and nuts, can vary greatly depending on the material used, as shrinkage characteristics differ among materials. Although the chosen materials allowed for a controlled comparison of the techniques, expanding the scope to include additional materials could provide valuable insights into their performance and accuracy. Future research could investigate the impact of various materials on the manufacturing process, leading to a more comprehensive understanding of their properties and applications. This approach would not only enhance the validity of the findings but also contribute to the ongoing advancement of additive manufacturing technologies. By examining a broader array of materials, optimal choices for specific applications could be identified, particularly in industries requiring precise and reliable components. The feedback regarding this limitation is appreciated and recognized as a vital avenue for future studies. The incorporation of diverse materials will undoubtedly enrich the field of additive manufacturing and its applications in mechanical engineering. Ultimately, the findings of this study pave the way for further research that addresses these aspects, promoting innovation and efficiency in manufacturing practices. Additive methods can be used for various screw connections. Given the variety of materials, designers can adapt them to different conditions. They are most suitable for static connections. Some prints have considerable strength properties but also exhibit significant brittleness, which could lead to fractures in dynamic connections. An exception might be dynamic connections with low forces.

## 5. Conclusions

Based on the conducted work and the analysis of the obtained results, several key conclusions can be drawn. Firstly, the use of a 3D scanner proved to be highly effective for the precise measurement of the surface characteristics of spline connection elements. This technology allowed for accurate data collection, facilitating a deeper understanding of the components’ geometry. Additionally, the implementation of a contour graph enabled the detailed registration of individual screw profiles. This method provided insights into the intricate shapes of the screws, contributing to the overall accuracy of the measurements.

When examining dimensional deviations for the spline connection components, specific ranges were identified. For the shaft, the deviations were noted to vary from −0.11 mm to +0.18 mm, indicating a relatively small but significant range of variation. In contrast, the sleeve exhibited a positive deviation of +0.24 mm, highlighting its unique dimensional characteristics. Furthermore, the study identified the PolyJet technique as the most accurate method for manufacturing screws. This technique surpassed others in terms of precision, suggesting its potential for high-quality screw production. Conversely, when it came to producing nuts, the SLA technique emerged as the most precise option available.

The research findings also demonstrated that the spline connection elements fit together effectively. This compatibility indicates that dimensional deviations resulting from the manufacturing process do not significantly affect the overall fit of the components. Moreover, the results confirmed that screw connection elements produced using different additive manufacturing techniques are compatible with each other. Such compatibility is crucial for ensuring functionality and ease of assembly in practical applications. Overall, the insights gained from this work contribute to a better understanding of the geometric accuracy of additively manufactured elements. This understanding can inform future research and applications in the field of additive manufacturing, particularly concerning spline connections and screw fittings.

## Figures and Tables

**Table 1:** FDM process parameters.

Filament Diameter [mm]	Nozzle Diameter [mm]	Table Temperature [°C]	Nozzle Temperature [°C]
1.75	0.4	60	230

**Table 2:** 3D ATOS Triple Scan II Blue Light parameters.

Scanner Parameter	Value
Cameras × pixels	2 × 5,000,000
Measured area	38 × 29–2000 × 1500 mm^2^
Point density	0.02–0.79 mm
Working distance	490–2000 mm
Number of points per scan	5,000,000
Work temperature	5–40 °C

**Table 3:** Dimensions of screws and nuts produced using various techniques and the reference element.

Screw and Nut	Screw Dimension [mm]	Nut Dimension [mm]	Distance Between Thread Grooves [mm]
Reference element	29.72	26.72	3.4952
PolyJet	29.73	26.32	3.5080
SLA	28.70	26.55	3.5174
FDM	29.52	26.44	3.5240

## Data Availability

Data are contained within the article.
